# App-based primary care in South Africa: A conceptual pathway from telemedicine service acceptance to patients’ continuance intentions

**DOI:** 10.4102/phcfm.v18i1.5191

**Published:** 2026-04-23

**Authors:** Grethe van Tonder, Christian D. Pentz, Ronel du Preez

**Affiliations:** 1 Department of Business Management, Faculty of Economic and Management Sciences, Stellenbosch University, Stellenbosch, South Africa; 2 Department of Industrial Psychology, Faculty of Economic and Management Sciences, Stellenbosch University, Stellenbosch, South Africa

**Keywords:** telemedicine service acceptance, healthcare, app-based primary care, South African public healthcare sector, patient participation, patient satisfaction, trust in telemedicine services, continuance intention

## Abstract

**Background:**

This study reports on the quantitative research phase of a mixed-methods study that investigated patients’ acceptance of an application (app)-based telemedicine service for primary care aimed at South African public healthcare sector patients.

**Aim:**

This study aimed to investigate the relationships between telemedicine service acceptance and eight antecedents of such acceptance, as well as the relationships between telemedicine service acceptance, perceived value, patient participation, patient satisfaction with a telemedicine service, patient trust in telemedicine services and two dependent variables, namely both patients’ continuance intentions towards a telemedicine service and towards a telemedicine service provider.

**Setting:**

The research was conducted in South Africa and focused on an app-based telemedicine service, Kena Health, a provider of app-based primary care at the time this research was conducted.

**Methods:**

Quantitative data were collected using a self-administered online questionnaire through the Qualtrics data collection platform. Partial Least Squares Structural Equation Modelling (PLS-SEM) was used to conduct the statistical analysis of a sample (*n* = 505) of respondents.

**Results:**

Statistically significant effects on patients’ telemedicine service acceptance were confirmed for perceived compatibility, innovativeness, privacy perception and care perception. All the hypothesised relationships between telemedicine service acceptance, perceived value, patient participation, patient satisfaction with a telemedicine service, trust in telemedicine services and patients’ continuance intentions towards the service and the telemedicine service provider were statistically significant.

**Conclusion:**

In South Africa, telemedicine services present a practical and scalable solution to more effectively address healthcare, particularly for underserved communities. This novel study offers much needed insights to improve healthcare delivery through digital innovation.

**Contribution:**

Two antecedents of telemedicine service acceptance that had not previously been considered in the technology acceptance theory, namely (positive) privacy perception and care perception, were confirmed. Insights are provided regarding the effect of patients’ trust in telemedicine services on their continuance intentions towards the service and the service provider under investigation.

## Introduction

A global surge in the use of telemedicine services is evident, especially since the early stages of the coronavirus (COVID-19) pandemic in 2020.^1^ Although telemedicine services were already being used before COVID-19 in several developed countries, the need for and relevance of telemedicine in developing countries, such as South Africa, was highlighted by the pandemic. Percept^2^ argues that telemedicine provision makes affordable healthcare services more readily accessible, especially for people who have difficulty accessing the necessary healthcare services because of logistical, geographical and financial constraints. In South Africa, telemedicine services focus on the medical needs of individuals who might not have medical insurance and who are typically reliant on the country’s overburdened public healthcare system.

Although South Africa is associated with providing high-quality healthcare on the African continent, the two-tiered healthcare system used in the country is regarded as highly unequal. The public healthcare sector (serving about 80% of the population) is underfunded, while most citizens are unable to afford the high costs of private medical care provided in the private healthcare sector.^3,4^ Furthermore, there is a vast disparity between public and private healthcare facilities in most parts of the country, and many of the leading medical specialists work in the private sector only.^5^ Based on the associated quality of service delivery in the private healthcare sector, people in South Africa are encouraged to opt for private medical insurance. This is so even though public healthcare services are offered at a reduced cost to make provision for citizens who earn the lowest levels of disposable income.^3,6^

Any individual in South Africa has access to the country’s public healthcare services, irrespective of their nationality or immigration status, but the public healthcare system primarily serves individuals who cannot afford private health insurance.^7,8^ The public healthcare sector is funded by the South African government that mainly uses taxation income and point-of-care fees by individuals who use public health services.^3,7^ Although the public sector healthcare services seem highly affordable to individuals with the lowest disposable income, the perceived total cost of these services can be high. The opportunity cost to consult a healthcare professional, such as travel and associated time costs, income loss given the absence from work, and the health risk of postponed medical care all add to the perceived total cost of the healthcare service.^9,10^

Serving approximately 20% of the population, the private healthcare sector in South Africa is much smaller than its public counterpart. Yet, the private health sector is regarded as comparable to healthcare delivery in developed countries such as Germany, the United Kingdom and France.^3^ About 79% of the doctors in South Africa work in the private sector in approximately 200 private hospitals across the country.^11^ However, the South African private healthcare sector has been criticised for being monopolised by a small number of prominent providers, and overpricing. This is so despite the fact that its services are generally regarded as of significantly higher quality than those offered by the public sector.^12^ With private healthcare insurance, patients can choose their own healthcare professional, access specialist healthcare without necessarily obtaining a general practitioner’s referral, and experience much shorter waiting times when making use of services in the private sector.^3^

According to Rensburg,^4^ inequality is perpetuated by the way in which the South African healthcare system is funded, therefore it is paramount to establish a strong network of well-trained and competent community health workers to provide primary healthcare. Because most public sector institutions in the country that provides primary care, especially in rural areas, are too poorly maintained and equipped to consistently provide efficient healthcare services, many South African citizens have lost trust in the public healthcare system.^9,13^ Naher et al.^14^ argue that a lack of good governance, mismanagement of resources, and corruption may be viable reasons for the poor maintenance of public health institutions by governments. Therefore, evidence from literature has put forward the use of alternative platforms or methods, such as mobile platforms, to provide the necessary healthcare to individuals who are typically reliant on these public health institutions for receiving medical care.^15^ In this respect, mobile phone usage in South Africa has posed an effective alternative platform to bridge the gap between quality primary care delivery and patients’ access to such care. It can also help to reduce the deficiencies in the already overburdened public healthcare system in South Africa.

At the time of this research, specific antecedents relevant to patients’ acceptance of telemedicine services in the South African public healthcare sector had not yet been identified. Furthermore, research on the possible subsequent relationships between telemedicine service acceptance and the relevant outcomes for telemedicine service providers remains limited. The aim of the study was to investigate the relationships between telemedicine service acceptance, and eight identified antecedents of such acceptance, as well as the relationships between telemedicine service acceptance, perceived value, patient participation, patient satisfaction with a telemedicine service, patient trust in telemedicine services and two dependent variables, namely both patients’ continuance intentions towards a telemedicine service and towards a telemedicine service provider.

### Theoretical underpinnings

The study applied to the service domain in business and marketing management and thus drew on theories fitting to services marketing, relationship marketing, customer relations, and consumer behaviour.

#### Service-dominant logic

In comparison to the goods-oriented view, the service-oriented view of marketing is customer-centric and market-driven, implying not only a customer-oriented approach to business practice and marketing but also an emphasis on collaboration with, and learning from, customers whereby the service adapts to customers’ individual needs and dynamics to define and co-create value.^16,17^ It is implied that the nature of the service interaction determines the success of the service provider. Furthermore, according to Vargo and Lusch,^17^ service-dominant logic alludes to a ‘sense-and-respond’ strategy to ensure the service provider’s success, rather than a ‘make-and-sell’ strategy. In the context of healthcare service delivery, these foundational principles of service-dominant logic play a crucial role.

#### Theories of reasoned action and planned behaviour

As the research focused on explaining consumer behaviour in a healthcare delivery context (more specifically, a virtual healthcare delivery context represented by a telemedicine service), the theories of reasoned action and planned behaviour were relevant. Having been applied extensively in consumer behaviour research, these theories provide a premise for understanding and predicting human behaviour across an array of different domains.^18,19,20^ Moreover, one of the focal points of the theories of reasoned action and planned behaviour is to explain the behavioural intentions of individuals in different consumption settings. In the case of this study, however, the focus was on investigating patients’ acceptance (which implies actual use) of a telemedicine service in a South African context, rather than on patients’ mere behavioural intentions (which cannot be assumed to imply actual use) towards such a service. As a result, it was possible to further explore the patients’ continuance intentions towards the service as possible behavioural outcomes after initial acceptance. Nonetheless, acknowledging and including the foundations of the theories of reasoned action and planned behaviour was still deemed applicable, as the research strongly leaned on the premises posed by the Technology Acceptance Model (TAM), which originated from the theories of reasoned action and planned behaviour.^21^

#### Eight antecedents of telemedicine service acceptance

In the first phase of this exploratory sequential mixed-methods study, eight antecedents of patients’ acceptance of an application (app)-based telemedicine service for primary care were identified through a thematic analysis of the primary qualitative data. These antecedents were included in the subsequent quantitative research phase, which is reported here (refer to H_a_^1^ – H_a_^8^ in Figure 2). For a detailed discussion of the preceding qualitative research phase, refer to Van Tonder et al.^22^

#### Telemedicine service acceptance

A telemedicine service is dependent on the relevant technology by which it is delivered to and used by patients. Therefore, the TAM formed the foundational and primary theoretical basis for this study’s investigation of patients’ telemedicine service acceptance.

According to Aminoff et al.,^23^ technology acceptance is an indicator of whether a certain new technology will ‘actually be used in a real-life setting’, which clearly implies that acceptance relates more to individuals’ actual use of a technology than their mere intention to use the technology. Similarly, Zhou et al.^24^ state that patients’ acceptance of telehealth systems (including telemedicine services) would always lead to actual adoption (usage) behaviour. In our study, patients’ telemedicine service acceptance was investigated and not their intention to use the service, because it cannot be assumed that intention to use necessarily implies that actual use has indeed taken place. Patients’ actual use (acceptance) was deemed necessary to explore a use experience to obtain evaluations of satisfaction, trust and continuance intentions.

Individuals’ acceptance of an innovation can be deemed a prerequisite for the adoption and diffusion of an innovation, which also applies to technology innovation.^25,26,27^ Thus, once again, acceptance reflects actual use given that (technology) innovation diffusion can only occur once innovators, early adopters, and early-majority individuals decide to use the innovation (or the service delivered by means of the technology innovation) and continue to use it.^25,28,29,30^

Against this background and in accordance with S-D logic, the following hypotheses were put forward:

H_a_^9^: A significant positive relationship exists between telemedicine service acceptance and perceived value.

H_a_^10^: A significant positive relationship exists between telemedicine service acceptance and patient participation.

#### Perceived value

Zeithaml^31^ first conceptualised perceived value as the extent to which a customer receives what they expected from the service relevant to, and in exchange for, what they had paid for – that is, the cost involved for the service delivery. Gu et al.,^32^ Karjaluoto et al.,^33^ Tran and Le^34^ and Del Mar Alonso-Almeida^35^ similarly describe perceived value as the overall assessment that a customer attaches to a service utility. This assessment is based on the customer’s perceptions of the extent to which their service expectations were met – that is, the perceived outcomes of the service interaction relevant to what was paid for the service interaction, which would likely affect satisfaction, or dissatisfaction, with the service interaction and outcome(s). Zeithaml^31^ further states that what constitutes value for customers is usually highly personal. Zeithaml’s^31^ notion suggests that, in the case of our study, patients’ perceived value of a telemedicine service would likely be influenced by different factors with different importance for each individual patient, implying that patients’ perceived value of the service would not be identical.

Following the theoretical development on the concept of value, Tran Le et al.^36^ and Tuan^37^ posit that perceived value may represent an ongoing interaction between a customer and the service offering under consideration, suggesting continuality. According to Tuan,^37^ perceived value is an ‘interactive relativistic preference experience’, which concurs with the S-D logic’s experiential nature and the premise that customers are value co-creators during service delivery interactions. Investigating perceived value is especially applicable when the goal is to understand consumer behaviour in e-service contexts.^33^ Perceived value was therefore distinctly relevant to our investigation of patient behaviour pertaining to a telemedicine service. Consequently, the following was hypothesised:

H_a_^11^: A significant positive relationship exists between perceived value and patient satisfaction with a telemedicine service.

#### Patient participation

Customer co-creation during service delivery implies a customer’s participation during the service interaction. Customer participation (or patient participation in healthcare) refers to the extent to which a customer actively engages in the co-creation and delivery of a service by contributing effort, information about their personal needs, and participation in decision-making processes and suggestions.^38,39,40,41^

Considering the context of app-based primary care telemedicine services, patient participation (following patient acceptance of the service and preceding patient satisfaction with the service) can be defined in accordance with mandatory customer participation. Both the presence of the patient (although virtual and not in person) and information-sharing are vital for the service to be delivered. Consequently, considering the insights provided by Tuan^37^ and the context of our study, patient participation was conceptualised as the patients’ engagement or involvement in the healthcare delivery process in its entirety, given that physical, virtual and mental resources are spent to participate in the service interaction. Patient participation typically relates to patients’ investment in time, knowledge, effort, and information-sharing in the healthcare service interaction, including other factors that may be cultural or social in nature, such as information-sharing on cultural practices regarding medical needs.^37^ The perceived level of participation necessary to be able to engage in the service interaction may differ from patient to patient.^37,40^ Therefore, patients would likely invest in different resources during the service interaction and exert different behaviours during their participation. As a result, different personal perceptions of service quality are formed, which implies that patients’ satisfaction or dissatisfaction with the service will also vary.^37,42,43^

Accordingly, the following hypothesis was considered:

H_a_^12^: A significant positive relationship exists between patient participation and patient satisfaction with a telemedicine service.

#### Patient satisfaction

It can be argued that, in a healthcare delivery context, satisfaction should not be assessed from a transaction-specific perspective, but from a cumulative perspective. The nature of receiving healthcare is highly personal. A patient’s satisfaction would likely be determined by the entire service delivery experience (starting at acceptance and ending with outcome evaluation), and not only by one specific interaction.^30,37^

A clear definition of patient satisfaction with telemedicine services still seems to be lacking, despite the fact that several prior studies investigated patient satisfaction with telemedicine services.^44,45,46,47,48,49^ The vastly different purposes of different telemedicine services (e.g. primary care compared to diabetes care or epilepsy care), suggest that different criteria would determine patient satisfaction, depending on the type of telemedicine service at hand.

Patient satisfaction was considered as following patients’ perceived value of the service and their participation during the service interaction. This inevitably implies more than the impact of a singular experience on a patient’s evaluations that would determine satisfaction with the service. In app-based telemedicine service delivery, both the patient’s interaction experience with the consulting healthcare professional and the patient’s experience of using the app (technological platform to provide and receive the service), as well as patients’ evaluations of the extent to which the service met their expectations, would cumulatively inform their satisfaction (or dissatisfaction) with the service.

Tuan’s^37^ definition of customer satisfaction in an e-services context was argued to adequately apply to the present study’s context, while also providing for the possible effect of both hedonic and utilitarian benefits on such satisfaction. Tuan^37^ proclaims that patient satisfaction with a telemedicine service is a ‘summated psychological state resulting from cognitive appraisal’ regarding patients’ perceived relationship between their (confirmed or disconfirmed) expectations of the service and their overall consumption or interaction experience throughout the entire service continuum.

Pertaining to the outcomes of patients’ potential satisfaction with a telemedicine service, support and evidence for the satisfaction-trust-continuance intention relationship in a service context were confirmed.^50^ Therefore, the following hypotheses were considered:

H_a_^13^: A significant positive relationship exists between patient satisfaction with a telemedicine service and continuance intention towards a telemedicine service.

H_a_^14^: A significant positive relationship exists between patient satisfaction with a telemedicine service and continuance intention towards the telemedicine service provider.

H_a_^15^: A significant positive relationship exists between patient satisfaction with a telemedicine service and trust in telemedicine services.

#### Trust in telemedicine services

When trust is present, it is implied that confidence in the exchange partner is also present. Trust represents the generalised expectancy of one party that another party’s word and integrity can be relied on.^51,52,53,54^ Trust in telemedicine services was considered as patients’ trust in telemedicine services in general, including trust in the providers of such services because, according to Van Velsen et al.,^55^ patients’ trust in telemedicine services would imply (and would be partially measured by) their trust in the telemedicine service provider involved. If trust in telemedicine services were to be absent, it could be assumed that trust in the telemedicine service provider would inevitably also be absent. As a result, general trust in telemedicine services was investigated as a probable outcome of patients’ satisfaction with the service^49,56^ and a possible preceding variable of patients’ continuance intentions towards both a telemedicine service and the specific telemedicine service provider involved.^47,57,58,59^ Consequently, the following hypotheses were formulated:

H_a_^16^: A significant positive relationship exists between trust in telemedicine services and continuance intention towards a telemedicine service.

H_a_^17^: A significant positive relationship exists between trust in telemedicine services and continuance intention towards a telemedicine service provider.

#### Patients’ continuance intentions

The Post-acceptance Model (PAM) by Bhattacherjee^58^ provided a premise for choosing two dependent variables as the proposed preferential behavioural outcomes of establishing service provider–patient relationships in a telemedicine service delivery context. Bhattacherjee^58^ showed that, based on the expectation-confirmation theory, people’s continuance intentions towards e-services would be significantly affected by their satisfaction with the e-service, following their initial acceptance of the e-service.

Similarly, Lu et al.^60^ define continuance intention as a person’s intention to continue using a service after their initial acceptance of the service, whereas Akter et al.^56^ assert that continuance intention can be viewed as synonymous with a trusting attitude towards the service, given the evidence of the strong direct effect of trust on continuance intention.

Consequently, in this study’s context, ‘continuance intention towards a telemedicine service’ was conceptualised as patients’ intentions to continue using a telemedicine service after their initial acceptance of the service. More specifically, ‘continuance intention towards a telemedicine service provider’ was deemed as patients’ intentions to continue to use a particular telemedicine service provider’s service offering (in this study’s case being Kena Health), despite the presence of available alternative providers of a similar service offering. Zhou et al.’s^24^ research guided our argument that patients’ continuance intentions towards a telemedicine service and a specific telemedicine service provider should not be investigated as one singular outcome variable, but rather as two independent entities. The reason for this approach is that a patient may perhaps be willing to continue using a specific type of telemedicine service, while potentially preferring to use a different service provider for future subsequent use of telemedicine services. This decision would be especially relevant if the patient experienced an unpleasant or dissatisfactory service interaction with the initial telemedicine service provider.

**FIGURE 1:**
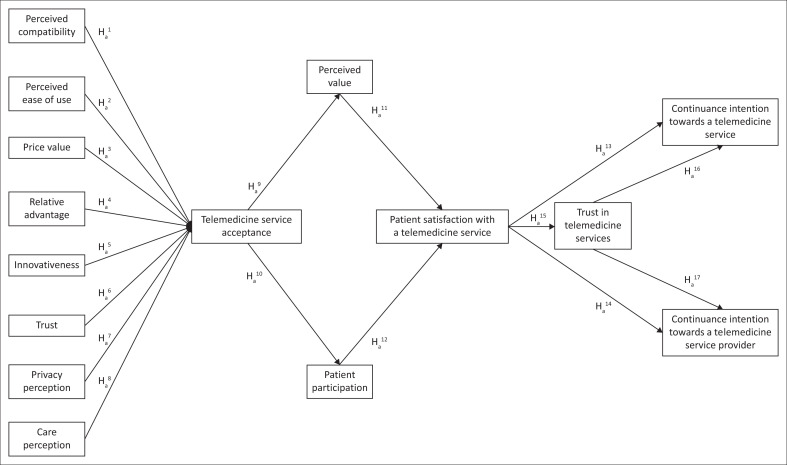
Research model.

In the light of the aforementioned discussion, Figure 1 depicts the research model according to which the research questions were investigated.

It should be observed that Figure 1 represents the research model for the quantitative measurement following the study’s initial qualitative research phase, in which the eight hypothesised antecedents of telemedicine service acceptance were identified through a thematic analysis of interview data (refer to Van Tonder et al.^22^).

## Research methods and design

### Study design

An exploratory sequential mixed-methods design was used. Please refer to Van Tonder et al.^22^ for a discussion of the results of the initial (qualitative) research phase. In the article presented here, the second (quantitative) research phase is reported, focusing on the statistical measurement of the relevant antecedents (as identified in the preceding qualitative research phase) of telemedicine service acceptance. Further relationships between telemedicine service acceptance, perceived value, patient participation, patient satisfaction with a telemedicine service, patient trust in telemedicine services and the two dependent variables, namely patients’ continuance intention towards both a telemedicine service and the telemedicine service provider, are also reported and discussed. These two dependent variables were considered as possible outcomes of patients’ satisfaction with and trust in the telemedicine service under consideration.

### Setting

The study was conducted in the context of Kena Health,^61^ a South African mobile app-based telemedicine service in primary care, whose service takes on the form of direct virtual provider–patient primary care delivery on a mobile application platform. The service includes consultation options according to patients’ preferences, such as a chat function, a voice call or a video call. Kena Health aimed to improve access to quality primary care by significantly lowering the cost of consultations with medical professionals (specifically nurses, general practitioners and mental health professionals) as patients were enabled to access medical care from their mobile phone, irrespective of their location. Both on-demand and scheduled consultations could be delivered, and patients had the choice of either consulting via text, phone call or video call through the Kena Health app. Kena Health’s target audience predominantly represents South African individuals who are reliant on the country’s public healthcare sector facilities to receive primary care because of a lack of medical insurance to be able to afford private healthcare.^61^ This target audience signified an ideal target population given that the research was specifically aimed at investigating app-based primary care in the context of the country’s public healthcare sector.

### Study population, sample size and sampling strategy

The target population of the study was South African adults (> 18 years) who had at least one prior exposure to or experience of the Kena Health app-based telemedicine service for primary care and who were on Kena Health’s client database up to January 2024.

The sample size of 505 responses was considered sufficient because Partial Least Squares Structural Equation Modelling (PLS-SEM) analyses generally do not need large samples. Moreover, we did not have control over the exact sample size as we were reliant on patients’ consent to participate in the research. The authors did not have direct access to Kena Health’s client base. The invitation to participate in the research, as well as the link to the online questionnaire, was distributed to Kena Health’s client base by a company representative. Therefore, we did not have the opportunity to do a random selection from the list of patients on the client base. The approach to sampling is therefore best described as convenience sampling.

It is important to observe that several patients on Kena Health’s client base who agreed to participate in the study had access to medical insurance at the time of the data collection. This meant that they were not necessarily public sector patients who cannot afford medical insurance to pay for private healthcare sector services. However, these patients (who were the minority in the sample) also used Kena Health’s app-based telemedicine service for its benefits compared to other in-person primary care alternatives. The fact that these patients formed part of Kena Health’s client base suggests that patients who have medical insurance should not be overlooked when considering app-based telemedicine alternatives for delivering primary care. We argue that, in a South African context, the nature of telemedicine alternatives often lends itself to addressing the needs of patients who are typically reliant on the public healthcare sector and who often struggle with gaining access to quality healthcare services. However, patients who can indeed afford medical insurance, but who can also benefit from the service that an app-based alternative provides, point to a valuable alternative target audience for telemedicine service providers. After the inclusion of patients who indicated ownership of medical insurance, the quantitative sample still predominantly represented public healthcare sector patients.

### Data collection

The primary data were collected using a self-administered online questionnaire on the Qualtrics survey platform, which comprised screening criteria and demographic questions and pre-determined scales from the literature to measure the constructs under investigation (please refer to Table 1-A1 and Table 1-A2 for a full referenced list of the pre-determined scales used). The questionnaire was piloted among the interview participants who consented to partake in the preceding qualitative research phase. These participants were asked to confirm or disconfirm the questionnaire’s perceived length, complexity and understandability.

### Data analysis

A PLS-SEM analysis was conducted on the collected questionnaire data after it had been extracted from the Qualtrics survey platform. The software used to conduct the analysis was R package ‘seminr’ version 2.3.6. An initial descriptive analysis of the data was conducted and reported in the form of demographic data characterised by frequencies and percentages. Because PLS-SEM is non-parametric, statistical significance (*p*-values of the hypothesised relationships) was determined by bootstrapping (1000 bootstrap replicates). The measurement model assessment addressed the internal consistency, convergent validity and discriminant validity of the data, while the structural model assessment addressed the assessment of collinearity, the assessment of the coefficient of determination (*R*^2^) and the assessment of path coefficients.

### Ethical considerations

Ethical clearance to conduct this study was obtained from Stellenbosch University Social, Behavioural and Education Research Ethics Committee (No. 26695).

## Results

A total of 1062 responses were collected, of which 505 could be used for the empirical analysis. The sample size (*n* = 505) was determined by the exclusion of responses that did not meet the inclusion criteria. Thus, respondents who indicated that they were not South African citizens, that they were not at least 18 years old at the time of the data collection, or that they did not have at least one prior use experience of the Kena Health app, were excluded from the empirical data analysis.

### Demographic profile

However, these patients (who were the minority in the sample) also used Kena Health’s app-based telemedicine service for its benefits compared to other in-person primary care alternatives. Even though these patients do not typically represent the South African public healthcare sector patients, they were still included in the target audience, and their insights and responses were included in the data analysis based on the exploratory nature of this study. The fact that these patients formed part of Kena Health’s client base suggests that patients who have medical insurance should not be overlooked when considering app-based telemedicine alternatives for delivering primary care. We argue that, in a South African context, the nature of telemedicine alternatives often lends itself to addressing the needs of patients who are typically reliant on the public healthcare sector and who often struggle with gaining access to quality healthcare services. However, patients who can indeed afford medical insurance, but who can also benefit from the service that an app-based alternative provides, points to a valuable alternative target audience for telemedicine service providers. After the inclusion of patients who indicated ownership of medical insurance, the quantitative sample still predominantly represented public healthcare sector patients. The sample predominantly comprised respondents between the ages of 20 years and 40 years, with most being between 20 years and 25 years old. The majority of the sample were female (81%), whereas male respondents made up 18% of the realised sample. This gender ratio mirrored the gender distribution of Kena Health’s complete client base at the time of the data collection. Most respondents resided in the Gauteng province of South Africa (48%), with the remainder distributed across South Africa as follows: Western Cape (16%), KwaZulu-Natal (8%), Limpopo and the Free State (both 6%), Mpumalanga and the Eastern Cape (both 5%), North West (4%) and Northern Cape (2%). With regard to the sample’s income distribution, most respondents (38%) indicated a ‘take home pay’ of less than R5000.00 (approximately $280.00) per month. Most respondents (34%) indicated that their highest level of education was Grade 12 (National Senior Certificate), whereas 43% of employed individuals worked for a salary or wages, and unemployed individuals in search of work comprised 36%.

### Reliability analysis

Cronbach’s alpha reliability analysis of the data yielded values between 0.83 and 0.96, which are considered well above the generally accepted threshold of 0.70. In addition, the composite reliability score (assessing internal consistency) and the average variance extracted (AVE) score (evaluating convergent validity) of each variable were calculated and assessed. The results are shown in Table 1.

**TABLE 1:** Reliability indices for the variables included in the measurement model.

Variable	Composite reliability	Average variance extracted	Cronbach’s alpha
Perceived compatibility	0.92	0.75	0.89
Perceived ease of use	0.92	0.80	0.87
Price value	0.92	0.80	0.88
Relative advantage	0.91	0.66	0.87
Innovativeness	0.91	0.71	0.86
Trust	0.91	0.72	0.86
Privacy perception	0.89	0.61	0.83
Care perception	0.97	0.76	0.96
Telemedicine service acceptance	0.92	0.75	0.89
Perceived value	0.95	0.83	0.93
Patient participation	0.94	0.79	0.91
Patient satisfaction with a telemedicine service	0.95	0.83	0.93
Trust in telemedicine services	0.94	0.77	0.93
Continuance intention towards a telemedicine service	0.95	0.87	0.92
Continuance intention towards a telemedicine service provider	0.95	0.86	0.92

The yielded AVE scores were all above the threshold of 0.50, confirming the convergent validity of the measurement model. Pertaining to the outer loading scores of the data, the measurement model indicated a reasonable degree of convergent validity. Subsequently, the data’s discriminant validity was assessed to establish the construct validity of the measurement model using the heterotrait-monotrait (HTMT) ratio of correlations.

### Discriminant validity

A maximum threshold of 0.90 for HTMT ratio values^62^ was used to assess the discriminant validity of the data, as well as the upper confidence interval limit that needs to be below 1. Discriminant validity was confirmed. The measurement model assessment confirmed adequate reliability and validity of the data to proceed with structural model assessment.

### Structural model assessment

A PLS-SEM analysis was used to assess the predictive capabilities of the structural model and the hypothesised one-directional relationships comprising the path model.^63,64^ The structural model consisted of the following variables: telemedicine service acceptance, perceived value, patient participation, patient satisfaction with a telemedicine service, trust in telemedicine services, continuance intention towards a telemedicine service and continuance intention towards a telemedicine service provider. Structural model assessment was conducted by means of three primary evaluation criteria, namely the coefficient of determination (*R*^2^), assessing collinearity, and evaluating the significance of the path coefficients.

The study yielded variance inflation factor (VIF) scores all below 5.00, confirming the absence of multicollinearity between the variables under investigation.^65,66,67^

#### Assessment of the coefficient of determination (*R*^2^)

Table 2 provides the coefficient of determination (*R*^2^) values of each of the variables comprising the structural model and indicates the assessment of each value.

**TABLE 2:** Coefficients of determination.

Variable	*R* ^2^	Assessment
Telemedicine service acceptance	0.70	Moderate
Perceived value	0.59	Moderate
Patient participation	0.49	Weak
Patient satisfaction with a telemedicine service	0.65	Moderate
Trust in telemedicine services	0.66	Moderate
Continuance intention towards a telemedicine service	0.67	Moderate
Continuance intention towards a telemedicine service provider	0.70	Moderate

Based on the yielded *R*^2^ values, the eight antecedents of telemedicine service acceptance explained 70% of the variance in telemedicine service acceptance. The remaining 30% of the variance in telemedicine service acceptance that was not explained by the preceding eight antecedents indicated that the research was not exhaustive.

Telemedicine service acceptance, furthermore, explained 59% of the variance in perceived value and 49% of the variance in patient participation. Although an *R*^2^ score of 49% is considered weak according to the rule of thumb,^68^ it is considered very close to 0.50. Therefore, it was deduced that telemedicine service acceptance still explained the variance in patient participation adequately to make inferences pertaining to marketing practice. Perceived value and patient participation together explained 65% of the variance in patient satisfaction with a telemedicine service, which in turn explained 66% of the variance in trust in telemedicine services. Together, patient satisfaction with a telemedicine service and trust in telemedicine services explained 67% of the variance in continuance intention towards a telemedicine service and 70% of the variance in continuance intention towards a telemedicine service provider. This result indicated that the dependent variables were predicted by their preceding variables to a moderate extent.

#### Assessment of the path coefficients and hypotheses

Table 3 portrays the empirical results of the quantitative research phase of the study, including the *p*-value and path coefficient score of each one-directional hypothesis in the research model. The significance or non-significance of the relevant alternative hypothesis is also indicated.

**TABLE 3:** Path coefficient statistics.

H_a_	Path		*p*-value	Path coefficient (*β*)	Significant
	From	To			
Support H_a_^1^	Perceived compatibility	Telemedicine service acceptance	0.021*	0.11	Yes
Reject H_a_^2^	Perceived ease of use	Telemedicine service acceptance	0.724	0.02	No
Reject H_a_^3^	Price value	Telemedicine service acceptance	0.646	−0.02	No
Reject H_a_^4^	Relative advantage	Telemedicine service acceptance	0.078	0.08	No
Support H_a_^5^	Innovativeness	Telemedicine service acceptance	0.001*	0.27	Yes
Reject H_a_^6^	Trust	Telemedicine service acceptance	0.827	−0.01	No
Support H_a_^7^	Privacy perception	Telemedicine service acceptance	0.001*	0.21	Yes
Support H_a_^8^	Care perception	Telemedicine service acceptance	0.001*	0.35	Yes
Support H_a_^9^	Telemedicine service acceptance	Perceived value	0.001*	0.77	Yes
Support H_a_^10^	Telemedicine service acceptance	Patient participation	0.001*	0.70	Yes
Support H_a_^11^	Perceived value	Patient satisfaction with a telemedicine service	0.001*	0.46	Yes
Support H_a_^12^	Patient participation	Patient satisfaction with a telemedicine service	0.001*	0.42	Yes
Support H_a_^13^	Patient satisfaction with a telemedicine service	Continuance intention towards a telemedicine service	0.001*	0.37	Yes
Support H_a_^14^	Patient satisfaction with a telemedicine service	Continuance intention towards a telemedicine service provider	0.001*	0.61	Yes
Support H_a_^15^	Patient satisfaction with a telemedicine service	Trust in telemedicine services	0.001*	0.81	Yes
Support H_a_^16^	Trust in telemedicine services	Continuance intention towards a telemedicine service	0.001*	0.48	Yes
Support H_a_^17^	Trust in telemedicine services	Continuance intention towards a telemedicine service provider	0.001*	0.26	Yes

* , Significant at the *p* < 0.05 level.

**FIGURE 2:**
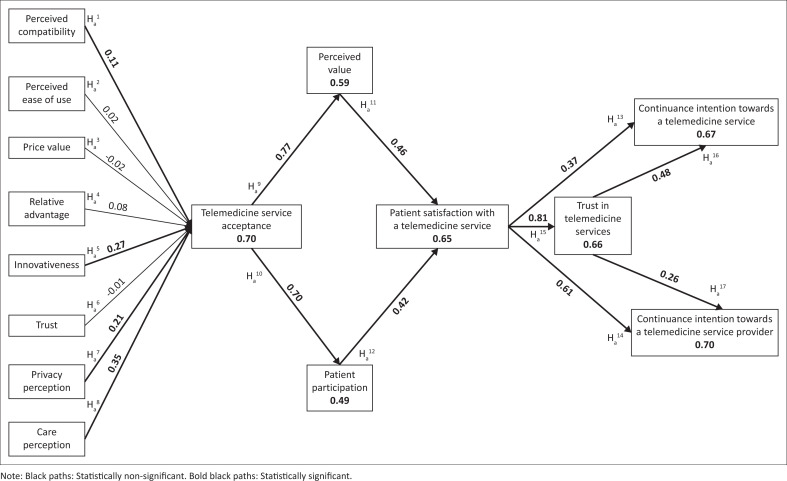
Path analysis of the research model.

Figure 2 displays the structural model, including the effects of the eight hypothesised antecedents of telemedicine service acceptance and shows the path coefficients and coefficient of determination (*R*^2^) scores as they pertain to each variable investigated in the research model. The statistically significant paths (indicated by the bold black lines) and the statistically non-significant paths (indicated by the regular black lines) can be distinguished.

## Discussion

Considering the eight hypothesised antecedents of telemedicine service acceptance (H_a_^1^–H_a_^8^) identified by the preceding qualitative research phase, four of the eight antecedents, namely perceived compatibility, innovativeness, privacy perception, and care perception (represented by H_a_^1^, H_a_^5^, H_a_^7^ and H_a_^8^) were confirmed as statistically significant antecedents of telemedicine service acceptance in the subsequent quantitative research phase, providing support for arguments in prior literature.^37,69^

It is therefore recommended that Kena Health prioritise strategies to increase perceived compatibility, innovativeness, privacy perception, and care perception among their target audience to strengthen the acceptance of their service. To increase patients’ innovativeness, it is suggested that a separate ‘how to’ tab on the Kena Health app is introduced, including video tutorials explaining best practices for utilising app-based consultations, as well as preparing adequately for a consultation (e.g. having the necessary information ready that the healthcare professional would most likely need, such as when symptoms were first experienced). Furthermore, to increase patients’ perceived compatibility of Kena Health’s service, we suggest that storytelling and testimonials by patients who have already made use of Kena Health’s service, and with whom the target audience would relate, be utilised on the brand’s media platforms. Such storytelling and testimonials could be powerful tools in providing evidence of Kena Health’s compatibility with the needs of patients representing different market segments. A ‘meet your doctor, nurse, mental health professional’ campaign consisting of short video testimonials could also be introduced to improve patients’ perceptions of privacy and care related to Kena Health’s services. The testimonials could portray Kena Health’s healthcare employees (who would be those directly consulting with patients), introducing themselves and sharing their values regarding their vocation. These values should relate to the employees’ treating patients’ vulnerability with genuine care and fulfilling each patient’s unique medical needs with the necessary privacy and respect.

This study made novel theoretical contributions by confirming privacy perception and care perception as newly identified antecedents of telemedicine service acceptance in the South African context (H_a_^7^ and H_a_^8^). Privacy perception and care perception have not yet been identified in prior literature as antecedents of technology acceptance applying to other technology acceptance contexts, such as telemedicine services and platforms. Care perception was also found to have the strongest effect on telemedicine service acceptance. This finding confirmed (to Kena Health specifically) the importance of prioritising strategies and tactics to establish, affirm and strengthen patients’ perceptions of genuine care associated with Kena Health’s service, especially given the contrast to what patients might have experienced with primary medical care at public healthcare alternatives.

Strategies to increase patients’ innovativeness, perceived compatibility, privacy perception and care perception can also contribute to patients’ increased perceived value and subsequent participation. The recommendation of these strategies is made in the light of the acceptance of H_a_^9^ and H_a_^10^ and provided that Kena Health ensures consistency in patients’ experience of sincere care and respect for their privacy throughout service interactions. The statistical significance of H_a_^9^ and H_a_^10^ provides support for prior research on perceived value and patient participation.^23,37^

Increasing and establishing patients’ satisfaction with the service by increasing patients’ perceived value is supported through the confirmed statistical significance of H_a_^11^ and provides agreement with prior literature.^37^ Consequently, it is suggested that Kena Health should consider the inclusion of a value-added service in addition to their existing service offering. For instance, Kena Health and similar app-based primary care telemedicine providers could encourage their healthcare employees to adopt a standard practice of following up with patients a week or two after a consultation took place to ascertain whether or not the patient scheduled a follow-up consultation. This action could even be performed informally, for example, by ‘checking in’ on the chat feature of the Kena Health app. For those patients who did not schedule a follow-up consultation themselves, such a strategy could affirm Kena Health’s genuine care for their clients and could create the perception that patients receive more from the service than what was originally paid for. As an incentive, these telemedicine providers could reward a healthcare employee for every 10 ‘unscheduled’ follow-up consultations conducted, whereas rewards for employees’ buy-in towards this strategy could include accumulations to their annual leave days or accumulations to a thirteenth pay-check at the end of the financial year.

Considering the significant positive relationship that was confirmed between patients’ participation in and satisfaction with the service (H_a_^12^), further support for prior research is provided, and it is consequently recommended that Kena Health consider providing patients with the option to join social networks or support groups in the Kena Health app, where patients can support one another.^37^ The sense of support, resources, and encouragement provided by such groups, as well as possible feelings of affirmed belonging, trust and safety that patients may perceive as a result, could motivate patients’ increased participation during service interactions and, consequently, contribute to increased satisfaction with Kena Health’s service. However, ensuring strict privacy measures pertaining to these groups (and reassuring patients of these measures) would be important, given that the likelihood of sensitive and personal information being shared during these group interactions would be high.

The results confirmed that patients’ satisfaction with the service should lead to an increase in patients’ continuance intention towards the type of telemedicine service delivered (H_a_^13^), continuance intention towards the telemedicine service provider, in this case being Kena Health (H_a_^14^), and trust in telemedicine services in general, of which the service offering forms part (H_a_^15^). The confirmed statistical significance of H_a_^13^, H_a_^14^ and H_a_^15^ provides support for arguments in prior literature concerning the effect of satisfaction on trust and continuance intention.^50,58^

To strengthen patients’ satisfaction and continuance intention towards an app-based primary care telemedicine service, we suggest that service providers place emphasis on their app-based telemedicine’s ability to fulfil individuals’ primary healthcare needs just as well as it would be with in-person primary care alternatives. This is expected to ensure a measure of patients’ satisfaction with the service outcomes necessary for further ensuring continuance intention towards app-based primary care telemedicine in general. To establish and increase patients’ continuance intention towards the telemedicine service provider (Kena Health), it is proposed that strategies are prioritised to personalise patients’ experience of Kena Health’s service. In this way, the service provider can be distinguished from other competing app-based telemedicine providers and can thus ensure a competitive advantage. For example, Kena Health could provide patients with the option to consult with a healthcare professional on the app in their mother tongue or a language in which the patient would feel most comfortable communicating. Pertaining to the South African context, where twelve official languages are spoken, such an option could motivate patients to continue using Kena Health’s service.

It is further recommended that Kena Health establish and increase patients’ trust in telemedicine services in general (resulting from their satisfaction with the service) by ensuring seamless coordination between providing the telemedicine service and in-person healthcare providers. Patients could, for instance, be provided with a list of physicians, specialists or pharmacists in their geographic area or be referred to a specific healthcare provider if it becomes clear during the patient’s consultation on the Kena Health app that the telemedicine service would not be able to provide the necessary solution on its own. Kena Health’s acknowledgement of where additional or alternative help is needed to ensure full recovery of the patient could reinforce patients’ trust in telemedicine services as a reliable way of receiving quality medical care.

Significant positive relationships were confirmed between patients’ trust in telemedicine services and their continuance intention towards the service – that is, towards app-based telemedicine (H_a_^16^) and towards the telemedicine service provider (H_a_^17^), respectively. The statistical significance of H_a_^16^ and H_a_^17^ supports Jiang and Lau’s^50^ research on the satisfaction-trust-continuance intention relationship.

To strengthen patients’ continuance intention towards app-based telemedicine by strengthening their trust in telemedicine services in general, we propose that Kena Health provide their clients with information on a continuous basis pertaining to how telemedicine (especially app-based telemedicine) is being integrated with various other medical fields, in South Africa, in particular, such as rehabilitation programmes, diabetes care and even surgery. This can be done by using Kena Health’s YouTube channel, and their Facebook, TikTok and Instagram platforms to provide patients with such information in an ‘easy to understand’ way, complemented by the captivating auditory and visual stimulation of video content. It is further recommended that Kena Health prioritise transparency not only in their marketing communication but also on their online platforms. Transparency should highlight what Kena Health specifically implements with regard to data handling practices, security protocols and policies, as well as the process implemented to ensure the delivery of quality, ethical healthcare. Transparency should further induce in patients a sense of trust and reliability associated with the kind of service delivered, which is expected to further increase patients’ continuance intention towards Kena Health, or the telemedicine service provider at hand.

The investigation of app-based primary care telemedicine service acceptance among patients in the South African public healthcare sector context makes a novel contribution to the literature. However, the results can only be applied to telemedicine services aimed at delivering app-based primary care. The findings only represent one telemedicine service provider’s client base, that is, Kena Health. As a result, the findings cannot be generalised to the entire South African population nor all South African public healthcare sector patients, or other app-based telemedicine service providers that do not offer app-based primary care specifically. Accordingly, a limitation is also posed regarding the size of the study sample. Furthermore, because the sample mainly represented participants (patients) who typically could not afford medical insurance and were therefore dependent on public healthcare sector services in South Africa, the results can only be applied to target markets with similar characteristics. Future studies can replicate this research among South African private sector patients, for example, those who have more disposable income and can thus afford medical insurance. These individuals may likely represent patients who possibly attach value to certain factors different to a public sector target market. Moreover, we suggest that similar research be conducted in the context of other telemedicine services, such as the treatment of specific conditions suitable for telemedicine care (e.g. diabetes and haemophilia) in a South African context, because the antecedents of the acceptance of such telemedicine services could differ from the antecedents of app-based primary care reported in this article.

We acknowledge that response bias during the data collection could have been present, as it is possible that respondents who decided to participate in the research potentially had a predominantly positive experience with Kena Health’s telemedicine service. Patients who had a predominantly negative service experience might have been less interested in participating in the research and are therefore less represented in the sample. However, the possibility of such response bias should not have influenced the data and results negatively, as the study was concerned with exploring factors that influenced patients’ acceptance of the service in the first place and how such acceptance translated into satisfaction and (positive) continuance intention towards the service.

## Conclusion

Among the eight investigated antecedents of telemedicine service acceptance, four of these were proven to have a statistically significant positive effect on telemedicine service acceptance. The remainder of the relationships under investigation between telemedicine service acceptance, perceived value, patient participation, patient satisfaction with a telemedicine service, patient trust in telemedicine services and both patients’ continuance intentions towards a telemedicine service and towards a telemedicine service provider were found to be statistically significant and positive.

This study confirms the value of remote consultations, diagnoses, and the monitoring and treatment of primary care needs of South Africans, especially in the light of an often mismanaged and overburdened public healthcare system. Moreover, the findings highlight that merely dealing with the physical medical needs of patients would not render telemedicine providers a competitive advantage. Rather, prioritising to remotely treat patients in a way that would honour their privacy, dignity and humanity – that is, treating the patient with the necessary empathy, respect and kindness – was found to be a distinct factor for current and future telemedicine service providers. This finding especially applies to primary care telemedicine providers who aim to attend to the medical needs of patients who are generally reliant on the South African public healthcare system if there were no telemedicine alternatives available. This study makes a case for the potential of a flourishing existence of app-based primary care service providers, such as Kena Health, in South Africa.

## Appendix 1

**TABLE 1-A1:** List of pre-tested scales for the hypothesised antecedents of telemedicine service acceptance.

Variable	Code	Theory and/or model	Scale item	Source
Perceived ease of use	PEU1	TAM	Learning to use telemedicine was not very difficult for me.	Kamal et al.^70^
	PEU2	TAM	I found it easy for myself to interact with healthcare professionals using telemedicine.	Kamal et al.^70^
	PEU3	TAM	Interacting with a telemedicine system was clear and understandable for me.	Kamal et al.^70^
Price value†	PV1	UTAUT 2	The telemedicine service was reasonably priced.	Venkatesh et al.^71^
	PV2	UTAUT 2	The telemedicine service was good value for money.	Venkatesh et al.^71^
	PV3	UTAUT 2	At the price I paid, the telemedicine service provided good value.	Venkatesh et al.^71^
Relative advantage‡	RA1	IDT	Telemedicine reduces medical errors.	Biruk and Abetu^72^
	RA2	IDT	Telemedicine facilitates diagnosis and treatment.	Biruk and Abetu^72^
	RA3	IDT	Telemedicine increases communication with healthcare providers.	Biruk and Abetu^72^
	RA4	IDT	Telemedicine can reduce the number of visits to healthcare centres, such as clinics.	Biruk and Abetu^72^
	RA5	IDT	Telemedicine enables me to fulfil my medical needs more quickly.	Biruk and Abetu^72^
	RA6	IDT	Telemedicine improves clinical decisions.	Biruk and Abetu^72^
	RA7	IDT	Telemedicine provides more comprehensive healthcare.	Biruk and Abetu^72^
Perceived compatibility‡	PC1	IDT	In my opinion, telemedicine is compatible with all aspects of my medical care needs.	Biruk and Abetu^72^
	PC2	IDT	Telemedicine is compatible with my current situation.	Biruk and Abetu^72^
	PC3	IDT	I think telemedicine fits well with the way I prefer medical care.	Biruk and Abetu^72^
	PC4	IDT	Using telemedicine fits well into the style of medical care I prefer.	Biruk and Abetu^72^
Trust (as antecedent of Acceptance)	TA1	Extended TAM	The telemedicine provider was trustworthy.	Jarvenpaa et al.^73^
	TA2	Extended TAM	The telemedicine provider wants to be known as one who keeps promises and commitments.	Jarvenpaa et al.^73^
	TA3	Extended TAM	I trust the telemedicine provider kept my best interests in mind.	Jarvenpaa et al.^73^
	TA4	Extended TAM	I didn’t find it necessary to be cautious with the telemedicine provider.	Jarvenpaa et al.^73^
	TA5	Extended TAM	The telemedicine provider had more to gain by delivering on their promises.	Jarvenpaa et al.^73^
	TA6	Extended TAM	The telemedicine provider met my expectations.	Jarvenpaa et al.^73^
	TA7	Extended TAM	The telemedicine provider cares about servicing a person.	Jarvenpaa et al.^73^
Privacy perception	PP1	Extended TAM	(Compared to in-person consultations), telemedicine would not collect too much information about a user.	Kuen et al.^74^
	PP2	Extended TAM	(Compared to in-person consultations), I would not be concerned about my privacy when using telemedicine.	Kuen et al.^74^
	PP3	Extended TAM	I have no doubts as to how well my privacy is protected while using telemedicine.	Kuen et al.^74^
	PP4	Extended TAM	My personal information would not be misused when using telemedicine.	Kuen et al.^74^
	PP5	Extended TAM	My personal information would not be accessed by unknown parties when using telemedicine in my everyday life.	Kuen et al.^74^
Care perception	CP1	(novel antecedent of technology acceptance)	The healthcare professional made me feel at ease.	Mercer et al.^75^
	CP2	(novel antecedent of technology acceptance)	The healthcare professional allowed me to tell my story.	Mercer et al.^75^
	CP3	(novel antecedent of technology acceptance)	The healthcare professional really listened.	Mercer et al.^75^
	CP4	(novel antecedent of technology acceptance)	The healthcare professional was interested in me as a whole person.	Mercer et al.^75^
	CP5	(novel antecedent of technology acceptance)	The healthcare professional fully understood my concerns.	Mercer et al.^75^
	CP6	(novel antecedent of technology acceptance)	The healthcare professional showed care and compassion.	Mercer et al.^75^
	CP7	(novel antecedent of technology acceptance)	The healthcare professional was positive.	Mercer et al.^75^
	CP8	(novel antecedent of technology acceptance)	The healthcare professional explained things clearly.	Mercer et al.^75^
	CP9	(novel antecedent of technology acceptance)	The healthcare professional helped me to take control of my health.	Mercer et al.^75^
	CP10	(novel antecedent of technology acceptance)	The healthcare professional made a plan of action with me concerning my health.	Mercer et al.^75^
Innovativeness	Inno1	TRI	I can easily understand new high-tech products/services (such as a telemedicine app) by myself.	Tuan^37^
	Inno2	TRI	I am usually the first one among my friends to acquire new high-tech products/ services (such as a telemedicine app).	Tuan^37^
	Inno3	TRI	I often keep up with the latest high-tech products/services in my areas of interest (such as a telemedicine app for receiving primary healthcare).	Tuan^37^
	Inno4	TRI	Other people come to me for advice on new high-tech products/services (such as a telemedicine app).	Tuan^37^

Note: Please see the full reference list of the article Van Tonder G, Pentz CD, Du Preez R. App-based primary care in South Africa: A conceptual pathway from telemedicine service acceptance to patients’ continuance intentions. Afr J Prm Health Care Fam Med. 2026;18(1), a5191. https://doi.org/10.4102/phcfm.v18i1.5191, for more information.

TAM, Technology Acceptance Model; UTAUT, Unified Theory of Acceptance and Usage of Technology; DT, Innovation Diffusion Theory; TRI, Technology Readiness Index.

† , Items adapted to address ‘the telemedicine service’ instead of ‘mobile internet’ for the purposes of the current study.

‡ , Items adapted to the perspective of the patient instead of the provider/medical professional

## Appendix 2

**TABLE 1-A2:** List of pre-tested scales for the variables in the research model forming the conceptual pathway between telemedicine service acceptance and the two dependent variables.

Construct	Code	Variable	Original item	Source
Telemedicine service acceptance†	ACC1	Acceptance	I think using telemedicine is a good idea for receiving medical assistance.	Zhou et al.^24^
	ACC2	Acceptance	I think using telemedicine protects my privacy.	Zhou et al.^24^
	ACC3	Acceptance	I think using telemedicine improves the convenience of receiving medical services.	Zhou et al.^24^
	ACC4	Acceptance	I think using telemedicine helps monitor my health.	Zhou et al.^24^
Perceived value‡	PVal1	Perceived value	Compared to the effort I need to make, the use of the telemedicine service is beneficial.	Tuan^37^
	PVal2	Perceived value	Telemedicine service is a good buy.	Tuan^37^
	PVal3	Perceived value	Compared to the time I need to spend, the use of the telemedicine service is worthwhile.	Tuan^37^
	PVal4	Perceived value	Overall, the use of the telemedicine service delivers me good value.	Tuan^37^
Patient participation§	PPar1	Participation	The healthcare professional acknowledged my opinion about treatment options.	Lankton and Wilson^76^
	PPar2	Participation	The healthcare professional encouraged me to ask questions.	Lankton and Wilson^76^
	PPar3	Participation	The healthcare professional explained all the treatment options to me so that I could make an informed decision.	Lankton and Wilson^76^
	PPar4	Participation	The healthcare professional strongly encouraged me to express all my concerns about the prescribed treatment.	Lankton and Wilson^76^
Patient experience††	PEx1	Patient experience	The healthcare professional saw me within a reasonable time.	Schumm et al.^77^
	PEx2	Patient experience	Things were explained to me in a way that was easy to understand.	Schumm et al.^77^
	PEx3	Patient experience	The healthcare professional listened carefully to me.	Schumm et al.^77^
	PEx4	Patient experience	It was easy to understand the information that the healthcare professional shared about my concerns.	Schumm et al.^77^
	PEx5	Patient experience	Respect was shown for what I had to say.	Schumm et al.^77^
	PEx6	Patient experience	The healthcare professional spent enough time with me.	Schumm et al.^77^
	PEx7	Patient experience	I would recommend the telemedicine service provider to others.	Schumm et al.^77^
Patient satisfaction with telemedicine service‡	S1	Satisfaction	I was pleased with the telemedicine service provided.	Tuan^37^
	S2	Satisfaction	The telemedicine service met my expectations.	Tuan^37^
	S3	Satisfaction	The telemedicine service provider is a good company to do business with.	Tuan^37^
	S4	Satisfaction	Overall, I was satisfied with the telemedicine service.	Tuan^37^
Continuance intention towards a telemedicine service‡‡	CIS1	Continuance intention towards a telemedicine service	My intention to continue using an app-based telemedicine service in general is high compared to using any alternative means (e.g., traditional in-person healthcare).	Lu et al.^60^
	CIS2	Continuance intention towards a telemedicine service	I will continue to use an app-based telemedicine service in general.	Lu et al.^60^
	CIS3	Continuance intention towards a telemedicine service	I will generally use an app-based telemedicine service on a regular basis in the future.	Lu et al.^60^
Continuance intention towards a telemedicine service provider§§	CIP1	Continuance intention towards a telemedicine service provider	My intention to continue using Kena Health is great compared to using any alternatives for primary care telemedicine.	Lu et al.^60^
	CIP2	Continuance intention towards a telemedicine service provider	I will continue to use Kena Health.	Lu et al.^60^
	CIP3	Continuance intention towards a telemedicine service provider	I will use Kena Health on a regular basis in the future.	Lu et al.^60^
Trust in telemedicine services¶¶	T1	Trust	I can trust telemedicine services.	Van Velsen et al.^55^
	T2	Trust	I can trust that possible problems with telemedicine services will be solved properly.	Van Velsen et al.^55^
	T3†††	Trust	I can trust telemedicine services more than other online services.	Van Velsen et al.^55^
	T4	Trust	I feel at ease when working with telemedicine services.	Van Velsen et al.^55^
	T5†††	Trust	I am comfortable providing my personal details for telemedicine services.	Van Velsen et al.^55^

Note: Please see the full reference list of the article Van Tonder G, Pentz CD, Du Preez R. App-based primary care in South Africa: A conceptual pathway from telemedicine service acceptance to patients’ continuance intentions. Afr J Prm Health Care Fam Med. 2026;18(1), a5191. https://doi.org/10.4102/phcfm.v18i1.5191, for more information.

† , Scale items address ‘telemedicine’ instead of ‘telehealth’ for the sake of consistency.

‡ , The words ‘service X’ in the original scale items were changed to ‘telemedicine service’ for the sake of consistency.

§ , Scale items address ‘the healthcare professional’ instead of ‘primary care physician’ for the sake of consistency; negatively phrased original items are stated positively for the sake of consistency in the measurement scale.

¶ , Original scales in the present tense were adapted to the past tense.

†† , Item wording was adapted in certain places to better fit the context of the study without changing the meaning of the original item.

‡‡ , Scale items were adapted to address ‘telemedicine services’ instead of ‘the website’ for the sake of consistency with the ‘trust in telemedicine services’ variable under consideration. Items were adapted to positive statements instead of negative statements for the sake of measurement consistency.

††† , Items originally stated in the negative were changed to the positive for the sake of measurement consistency.
